# Transcript expression plasticity as a response to alternative larval host plants in the speciation process of corn and rice strains of *Spodoptera frugiperda*

**DOI:** 10.1186/s12864-017-4170-z

**Published:** 2017-10-16

**Authors:** Karina Lucas Silva-Brandão, Renato Jun Horikoshi, Daniel Bernardi, Celso Omoto, Antonio Figueira, Marcelo Mendes Brandão

**Affiliations:** 1 Laboratório de Melhoramento de Plantas, Centro de Energia Nuclear na Agricultura, Universidade de São Paulo, Campus “Luiz de Queiroz”, Av. Centenário, 303, Piracicaba, SP 13400-970 Brazil; 2 Present address: Centro de Biologia Molecular e Engenharia Genética, Universidade Estadual de Campinas, Av. Cândido Rondon, 400, Campinas, SP 13083-875 Brazil; 3 Programa de Pós-graduação em Entomologia, Departamento de Entomologia e Acarologia, Escola Superior de Agricultura “Luiz de Queiroz”, Universidade de São Paulo, Av. Pádua Dias, 11, Piracicaba, SP 13418-900 Brazil; 4 Departamento de Entomologia e Acarologia, Escola Superior de Agricultura “Luiz de Queiroz”, Universidade de São Paulo, Av. Pádua Dias, 11, Piracicaba, SP 13418-900 Brazil; 5 Centro de Biologia Molecular e Engenharia Genética, Universidade Estadual de Campinas, Av. Cândido Rondon, 400, Campinas, SP 13083-875 Brazil

**Keywords:** Digestive enzyme, Ecological speciation, Fall armyworm, P450, Transcriptome

## Abstract

**Background:**

Our main purpose was to evaluate the expression of plastic and evolved genes involved in ecological speciation in the noctuid moth *Spodoptera frugiperda*, the fall armyworm (FAW); and to demonstrate how host plants might influence lineage differentiation in this polyphagous insect. FAW is an important pest of several crops worldwide, and it is differentiated into host plant-related strains, corn (CS) and rice strains (RS). RNA-Seq and transcriptome characterization were applied to evaluate unbiased genetic expression differences in larvae from the two strains, fed on primary (corn) and alternative (rice) host plants. We consider that genes that are differently regulated by the same FAW strain, as a response to different hosts, are “plastic”. Otherwise, differences in gene expression between the two strains fed on the same host are considered constitutive differences.

**Results:**

Individual performance parameters (larval and pupal weight) varied among conditions (strains vs. hosts). A total of 3657 contigs was related to plastic response, and 2395 contigs were differentially regulated in the two strains feeding on preferential and alternative hosts (constitutive contigs). Three molecular functions were present in all comparisons, both down- and up-regulated: oxidoreductase activity, metal-ion binding, and hydrolase activity.

**Conclusions:**

Metabolization of foreign chemicals is among the key functions involved in the phenotypic variation of FAW strains. From an agricultural perspective, high plasticity in families of detoxifying genes indicates the capacity for a rapid response to control compounds such as insecticides.

## Background

The intimate interaction between herbivorous insects and their host plants has an enormous influence on the dynamics and evolution of their populations. First, the ability to feed on plants has driven diversification and speciation processes in insects [1, 2]. Populations of oligophagous or polyphagous insects associated with host plants belonging to the same plant family or to different families, respectively [3], can become isolated into distinct lineages in response to spatial or temporal preferential use of hosts [4–6]. Should assortative mating of insects on the preferential host plant occur, pre-zygotic isolation may arise [7]. In short, the initial divergence due to host-plant preference can lead to speciation within insect lineages.

The study of ecological speciation – that is, adaptive divergence between populations due to ecological factors [7, 8] – encompasses the investigation of several mechanisms through which divergent selection can cause reproductive isolation [7, 9, 10]. The process of lineage differentiation is continuous and can lead to increasing reproductive isolation [5], designated as a “stage” of speciation by some authors [10].

How does the initial diversification arise in divergent populations that use alternative host plants? Recent studies have emphasized the role of phenotypic plasticity in diversification and speciation events, including numerous species of phytophagous insects that use different host plants [11–14]. If the initial preference for a new host plant is a plastic response, in the sense of the ability of a single-genotype organism to react to a novel environment and to produce different phenotypes [11, 13], the evolutionary mechanism behind the use of alternative host plants in the distributional range of an insect, leading to an increase in reproductive isolation, can follow the developmental-plasticity hypothesis proposed by West-Eberhard [12]. According to this model, adaptive selection of plastic phenotypes occurs in two steps: 1) a novel environmental factor affects plastic phenotypes, leading to novel variants; 2) novel variants affected by environmental recurrences of the initial stimulus are selected to produce evolutionary genetic change that can finally become fixed by genetic assimilation [13]. In this way, phenotypic plasticity can facilitate evolutionary change and speciation by enabling subsequent adaptation [13]. Accordingly, phenotypic plasticity allows diverse environments to perform a dual role in evolution, since it generates phenotypic variation that can potentially respond to selection. In addition, plasticity in one trait can influence the variation and selection in linked or correlated traits [11].

Another important matter related to divergent selection and the speciation process in the interactions between phytophagous insects and their host plants is whether adaptation is the product of many loci with small effects or of a few loci with a large effect [15]. A prevalent idea is that adaptation to a new environment, or to a host plant in the present scenario, would involve a few key loci with large effects on the organism genome [16, 17]. How then can we identify the key genes involved in the speciation process as a response to host-plant preference?

Our main purpose here was to identify plastic and evolved genes that are involved in ecological speciation toward primary and alternative host plants, attempting to answer how the host plants influence the differentiation of lineages with increasing reproductive isolation in polyphagous insect populations. The fall armyworm (FAW), the noctuid moth *Spodoptera frugiperda* (J. E. Smith), is an appropriate model to answer these questions. *S. frugiperda* is a polyphagous species and one of the most important pest in several crops worldwide, including corn (*Zea mays* L.), rice (*Oryza sativa* L.), and, more recently, cotton (*Gossypium hirsutum* L.). The most impressive feature of *S. frugiperda*, however, is its differentiation into host-plant-related strains, one that feeds preferentially on corn, cotton, and sorghum (corn strain, CS), and another that is found usually on rice and several pasture grasses (rice strain, RS) [18]. This distinction is indeed an ongoing process, as suggested by evidence of hybridization between the strains [19]. Several terms have been used in the literature to describe these strains, sometimes as synonyms in the same manuscript, such as biotypes [20], host strains [18, 19, 21–26], host races [19], host assemblages [26], ecological races [21], genetically differentiated forms [26], host forms [21, 27], genetic groups [19], and even sibling species [18, 21]. Numerous pieces of evidence confirm the differentiation of FAW into strains, including habitat, temporal and behavioral isolation, unidirectional mating (revised as pre- and post-zygotic barriers in [22] and in [19]). Although morphologically identical, the two strains possess at least two main reproductive isolation mechanisms that are responsible for their maintenance: differences in the composition of female pheromones [23], and in the period of reproductive activities [25]. Although there is no strong evidence of complete reproductive isolation between the strains [28], interbreeding between them results in loss of fertility [29].

The effect of host plants on the differentiation of these FAW strains has long been recognized [19], but how much the preferential use of a host plant contributes to reproductive isolation between the strains is still undetermined. Likewise, the genetic mechanism of adaptation and the preferential use of host plants in the speciation process of FAW are unknown. In agreement with the hypothesis that a few genes with key functions should be responsible for the adaptation to one host plant as opposed to another [16], few AFLP loci specific to each strain have been identified [20, 26].

Currently, the most commonly applied next-generation sequencing technique is transcriptome characterization, although few studies have used this approach to study the interactions between phytophagous insects and their host plants [30, 31]. Unbiased studies of the transcriptional-profile resulting from RNA-sequencing (RNA-Seq) are a powerful tool for studying the adaptation and speciation processes in such interactions, since they allow differentially regulated genes to be functionally characterized under diverse conditions. They are also the initial stage in understanding genetic, ecological and evolutionary mechanisms involved in the speciation process as a result of adaptive divergent selection in response to host-plant use [15, 31]. In an agricultural context, the mechanisms of insect adaptation to different alternative hosts offer a useful model for understanding how evolution may proceed in genetically engineered crops [32]. We applied RNA-Seq and transcriptome characterization to evaluate unbiased genetic-expression differences in larvae of the two strains of *S. frugiperda* fed on primary (corn) or alternative (rice) host plants. We consider corn as the primary FAW host plant because currently the species is more easily found on this host, while it is rarely found on rice in Brazil; however, the preference between these two host plants is considered ambiguous for the FAW [33].

According to the West-Eberhard model [12], the genes that underlie a plastic response to new environments, exhibiting differences in expression, can be the same genes that are differently regulated in recently diverged lineages [34]. Thus, we consider that genes that are differently regulated by the same FAW strain in response to exposure to different host plants, to be “plastic” in the sense that they are a reaction of the organisms to two distinct environments. Otherwise, differences in gene expression in the two strains fed on the same host plant are considered constitutive differences, and should be involved in the process of speciation between FAW strains.

We can consider that host-plant recognition and use-induced phenotypic changes involve multiple regulatory genes and processes through different hierarchies, as in other environment-induced phenotypic changes [13]. For this reason, an unbiased approach to estimate the gene expression is not only desirable, but also essential to better understand the phenotypic and genetic responses of phytophagous insects to preferential and alternative host plants, and how reproductive isolation evolves in these circumstances. Therefore, we first evaluated differences in the larval performance of the FAW strains fed on primary and alternative host plants under laboratory conditions. We then assembled and functionally characterized the unbiased transcriptome profile of *S. frugiperda*, with emphasis on differentially regulated genes in larvae reared in different conditions. Finally, we investigated the hypothesis that differentially regulated genes of the transcriptional plastic response to host plants are highly represented as constitutive differentially regulated genes in each FAW strain reared on different hosts.

## Methods

### Insect rearing

Populations of *Spodoptera frugiperda* for either CS or RS were kept on a white bean-based artificial diet [35] under laboratory conditions. The rice strain (RS) colony was originated from a cornfield collection of 170 larvae at Santa Helena de Goiás, Goiás, Brazil, in 2011-winter season. This population was separated in single pair mating in laboratory and the adults were genotyped to determine the strain. A single couple was genotyped as RS and the colony was established. The colony was maintained on artificial diet in mass mating cages for 2 years. In 2013, we collected insect samples from this population and established single-pair matings that were strain genotyped for the present study. The corn strain (CS) was obtained from a cornfield collection of 317 larvae at Campo Mourão, Paraná, Brazil in 2013-winter season. The population was kept in artificial diet and mass mating cages under laboratory conditions for ~ 5 generations. Then, we established single pair matings and genotyped both male and female to check the strain.

For each strain, one couple was placed in a cylindrical plastic cage (23 cm height × 10 cm diameter) for mating, and immediately after the female oviposited, the adults were removed from the cage to extract DNA for strain genotyping, as explained below. Experiments with both CS and RS were conducted with sibling larvae from the same family line.

One hundred twenty-eight to 144 neonate larvae of CS and RS (Table 1) were transferred to individual plates with a white bean-based artificial diet or with fresh leaves of one of the two host plants: i) corn (*Z. mays*), cultivar 2B688 Dow AgroSciences, hereafter referred to as the primary host plant; and ii) rice (*O. sativa*), cultivar Puitá INTA-CL, hereafter the alternative host plant. The six combinations were: 1) CS on diet; 2) RS on diet; 3) CS on corn leaves; 4) CS on rice leaves; 5) RS on corn leaves; and 6) RS on rice leaves. The leaves were replaced with fresh leaves every two days throughout the larval development period. The larvae were kept in climate-controlled chambers at 27 ± 1 °C, relative humidity of 60 ± 10% and a 14-h photophase. Part of the larvae were reared until pupation, and some 5th-instar larvae were stored in RNAlater® (Life Technologies, Carlsbad, CA, USA) for RNA extraction, in the proportion of 1:5 of mass:RNAlater (Table 1).

**Table 1:** Number of replicates in each larval stage during rearing on primary and alternative host plants

Feed Condition	N of 1st instar larvae	N of 10-day Larvae	N of 24-h Pupae*	N of Emerged Adults	Larval Developmental Time (Days) (Min-Max)
CS on corn	128	115	65 (43)	55	14.2 (12–17)
CS on rice	144	117	33 (46)	23	21.0 (18–25)
CS on diet	144	123	72 (46)	57	18.7 (16–24)
RS on corn	128	73	20 (40)	20	14.3 (14–18)
RS on rice	144	68	8 (48)	2	22.6 (20–25)
RS on diet	144	105	30 (52)	25	19.7 (17–25)

* Numbers in parentheses indicate 5th or 6th instar larvae removed before pupation for RNA extraction

### Strain genotyping

Total genomic DNA was extracted from each couple (both male and female) presumed to be of either CS or RS. DNA was extracted using the DNeasy Blood & Tissue Kit (Qiagen, Dusseldorf, Germany) from the thoracic tissue of each individual. The mitochondrial gene cytochrome c oxidase I (*COI*) (ca. 569 bp) was amplified using the primers JM76 and JM77, under the same PCR program described in [36]. Amplification reactions were conducted with 25 μL total volume, using 1 μL of total DNA, 2 mM of 25 M MgCl_2_, 40 μM of dNTPs, 0.2 μM of each primer, 1 U of GoTaq DNA Polymerase (Promega, Madison, WI, USA), 10% of 10X Taq buffer and 10% volume of 5% dimethyl sulfoxide (DMSO). PCR products were run in 1% agarose gel in TAE 1X buffer (40 mM Tris, 20 mM acetic acid, 1 mM EDTA (pH 8.0)) to confirm amplification. After amplification, 1.0 μL of FastDigest *MspI* (Thermo Scientific, Waltham, MA, USA) was added to 10 μL of each reaction, incubated at 37 °C for 10 min, and the complete volume was loaded in 2% agarose gel in TAE buffer.

A repeated DNA sequence known as FR [37] was also amplified for each male and female to confirm their strains, using primers FR-c and FR-2, and conditions described elsewhere [36], using the same PCR procedure as above. PCR products were run in 2% agarose gel in TAE buffer to observe the band patterns related to each strain.

### Larval performance

Larval performance in each rearing condition was evaluated by measuring the weight (in mg) of 10-day-old larvae and 24-h-old pupae, and the time (in days) to complete larval development (from first instar to pupa). Statistical differences were evaluated by log-transforming weight values and comparing mean differences among treatments, using the Tukey test [38].

### RNA extraction, library preparation, and sequencing

Twelve larvae from each feeding condition in the early 5th instar were stored in RNAlater. Later, larvae were removed from the storage reagent, their gut contents were washed with 0.9% NaCl physiological solution to remove residual food, and the whole bodies were immediately immersed in liquid nitrogen and ground together. To increase the power of the post-sequencing statistical analyses and efficiently use sequencing resources [39, 40], three independent biological replicates for each condition, with 12 larvae each, were conducted using this procedure, totaling 36 individuals from each treatment. Individual replicates were stored at −80 °C until RNA extraction.

RNA was extracted using TRIzol™ (Life Technologies) combined with Direct-zol™ RNA MiniPrep (Zymo Research, Irvine, CA, USA). Each sample was eluted in 40 μL of Ultrapure water (Qiagen). RNA quality was evaluated using a Nanodrop UV spectrophotometer (Techno Scientific, Wilmington, DE, USA), and quantified in a Qubit® 2.0 Fluorometer (Life Technologies). RNA validation was completed in an Agilent 2100 Bioanalyzer (Agilent Technologies, Santa Clara, CA, USA) in the Central Laboratory of High Performance Technologies (LaCTAD), University of Campinas, Brazil, where libraries were prepared using the Denaturing and Diluting Libraries for the HiSeq® and GAIIx (using 15 pM of sample per lane), TruSeq® RNA Sample Preparation v2, and cBot (Illumina, San Diego, CA, USA). Each of the three biological replicates per condition (library) was run in a different lane in an Illumina HiSeq 2500 System (Illumina), with 12 libraries per lane (Table 2).

**Table 2:** De novo assembly descriptive metrics

Parameters	
Number of contigs	71,425
Total size of contigs	66,937,894
Longest contigs	12,267
Shortest contigs	199
Mean contig size	937
contigs %A	28.65
contigs %C	20.15
contigs %G	20.25
contigs %T	28.66
contigs %N	1.99
contigs %non-ACGTN	0.30
Number of contigs non-ACGTN nt	200,604

### Transcriptome assembly

Illumina reads from all replicates were processed using Illumina pipeline v. 1.8 or later. Prior to assembly, reads from all replicates were merged into a single dataset and the resulting FASTQ files (Illumina) were filtered to remove low-quality bases (score < 30) and adapters, using the SeqyClean pipeline (https://github.com/ibest/seqyclean). The remaining data were normalized by a single-pass digital normalization using the “normalize_by_kmer_coverage” procedure of the Trinity assembler suite [41]. A de novo hybrid assembly using ESTs data from the SpodoBase project (http://bioweb.ensam.inra.fr/spodobase/) and in-house Illumina sequencing reads was performed with the MIRA assembler v. 4.9.3 [42] with two passes and the following settings: i) spoiler detection on (−AS:sd = yes), ii) 70% relative percentage of exact word matches (−SK:pr = 70), iii) maximum megahub ratio = 1 (−SK:mmhr = 1), iv) stepping increment = 2 (−SK:kss = 2), v) quality clipping on (−CL:qc = yes), vi) minimum base quality = 5 (−CL:qcmq = 5), vii) length of window for quality clipping = 5 (−CL:qcwl = 5), and viii) elimination of sequences that form a contig with <3 reads (−AS:mrpc = 3). The final transcriptome was constructed by filtering the CAF (Common Assembly Format) file generated by MIRA for contigs larger than 200 bases, which shows more than five times coverage. Chimeric assembled contigs were identified and removed using a suite of customized Perl script (blast CHECK).

### Annotation

Gene ontology (GO) annotation and sequence descriptions were indicated by a multi-step process, using a customized set of Perl scripts and local databases constructed with publicly available data (this database is available upon request to the authors). All assembled contigs were searched by similarity against NCBI REFSEQ [43] (updated on September 3, 2015) and MEROPS v. 9.12 [44], a specific database for peptidases, using an e-value cut-off of 10e^−5^ and HSP similarity threshold of 80%. Patterns of RNA families were indicated by HMMScan [45] using the RFAM database v. 12 [46]. Patterns of protein families from the PFAM database [47] were proposed by HMMScan using a set of translated peptides from candidate coding regions within the assembled transcriptome sequences indicated by transdecoder (https://transdecoder.github.io/).

The sequence description was achieved by integrating all database searches. Blast best hit results, from both databases previously described, were designated by a restrictive e-value and HSP similarity cut-off (1e^−10^ and 90% respectively) sorted by the latter; RNA and protein families from HMMScan were filtered by the Expectation Value (1e^−10^) in the “full sequence” column from the resulting analyses. Sequences that did not recover any information from all the databases used were tagged as “UNKNOWN DESCRIPTION”. The sequence description can be presented by all or any information from each of the databases. The gene ontology controlled vocabulary terms were assigned to all sequences by a custom Perl script that searches the GO local database (updated on September 5, 2015) using all sequence-similarity results from previous described searches, following the thresholds described above and removing all obsolete terms and redundant ontologies for the same sequence.

### Differential regulation in *S. frugiperda* strains feeding on primary or alternative host plants

Differential expression analyses were carried out with scripts from the RSEM pipeline [48] to prepare the transcriptome reference index and to calculate the relative expression within each library. Differential gene expression analysis was conducted using edgeR [49] with TMM normalization [50], as suggested in the Trinity pipeline procedure [41], and the *p*-values were corrected for multiple testing by the false discovery rate [50]. The RPKM values for the most differentially regulated genes (corrected p-value <0.001 and log_2_ fold change >2 or < −2) were submitted to a MySQL database for faster consulting and comparisons among conditions. Differential gene regulation was compared in four tests: two comparisons were run to test the response of each strain to the same primary or alternative host plant (CS on corn vs. RS on corn, and CS on rice vs. RS on rice) (constitutive response), and two comparisons tested the response of the same strain to primary and alternative host plants (CS on corn vs. CS on rice, and RS on corn vs. RS on rice) (plastic response).

To investigate the hypothesis that differentially regulated genes of a transcriptional plastic response to host plants are also highly represented as differentially regulated evolved genes, we surveyed contigs that were simultaneously differentially regulated in both response types. First we constructed two lists of differentially regulated genes, the first composed of genes present in the two plastic comparisons, and the second composed of genes present in the two constitutive comparisons. Then we examined whether overregulated genes in the first list were also present in the second one. These contigs were gathered by a set of SQL scripts, consulting the previously generated database, and gene ontology annotation was summarized using REVIGO [51]. The 10 most enriched GO terms were identified by the weight01 method implemented in topGO [52].

All scripts used in this study are available upon request to the authors.

## Results

### Strain genotyping

The identity of each parental couple was confirmed as CS and RS by both the PCR-RFLP analysis of the mitochondrial *COI* gene and the FR repeated DNA sequence (data not shown).

### Larval performance

**Fig. 1:**
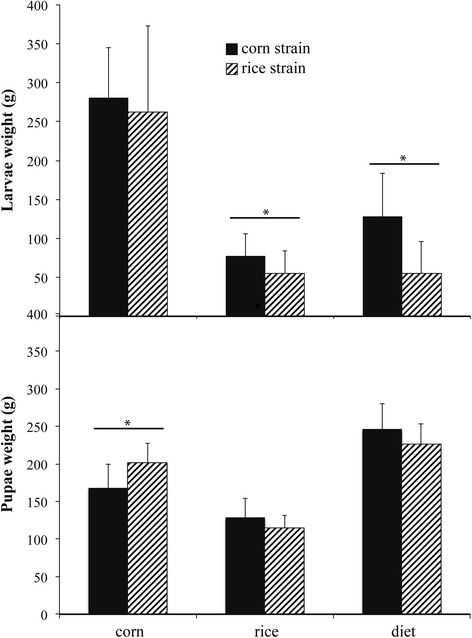
Larval performance described as 10-day-old larval weight (above) and 24-h-old pupal weight (below) in CS and RS reared on corn and rice leaves and on an artificial diet. * indicates significant difference (*P* < 0.05) between strains reared in the same conditions

Larval weight at 10 days was similar in both CS and RS when they were reared on corn leaves (Fig. 1). CS larvae were significantly heavier than RS larvae when they were fed on rice and on the artificial diet. However, the pupal weight was not significantly different for CS and RS fed on rice and on the artificial diet, but RS pupae were significantly heavier than CS pupae when they were fed on corn leaves. Although the larval and pupal weights did not differ significantly between the two strains, RS larvae required at least two more days to pupate than CS larvae when both were reared on rice leaves, indicating phenotypic differences between CS and RS as a response to the same host plant (Table 1). Larval development time ranged from 12 to 25 days, depending on feeding conditions (Table 1). Both CS and RS showed the longest larval development times when reared on the alternative host plant (rice leaves), and the shortest times on the primary host plant (corn leaves).

### RNA sequencing

As expected, the number of reads varied among replicates. However, the quality of sequencing did not vary widely, with more than 80% of the reads with a quality index above Q30.

### Transcriptome assembly and annotation

**Fig. 2:**
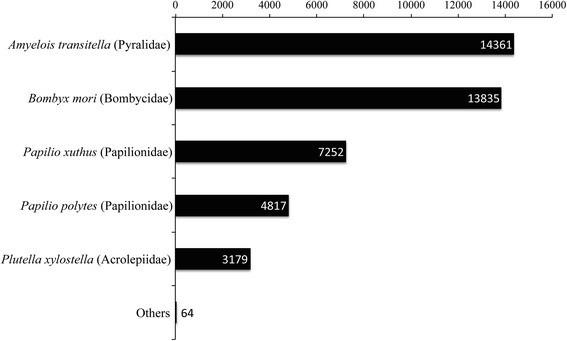
Contribution of species of Lepidoptera for annotation. The number of annotated contigs is noted on the bars

After assembly, 71,425 contigs, with a mean size of 937 nt, were annotated by similarity (Table 2), and 26,389 contigs (ca. 37%) resulted in an unknown annotation. The size of non-annotated contigs varied from 199 to 7950 nt (mean size 695 nt). Most of the annotation information (98.6%) came from the phylum Arthropoda, class Insecta. The order Lepidoptera represented the source of annotation for 98.9% of the contigs within Insecta, but other orders were represented as well in lower numbers, including Hymenoptera, Diptera, Hemiptera, Coleoptera, and Phythiraptera. Within the order Lepidoptera, the pyralid *Amyelois transitella* was the most represented species (33%), followed by *Bombyx mori* (31.8%) and *Papilio xuthus* (16.7%) (Fig. 2).

**Fig. 3:**
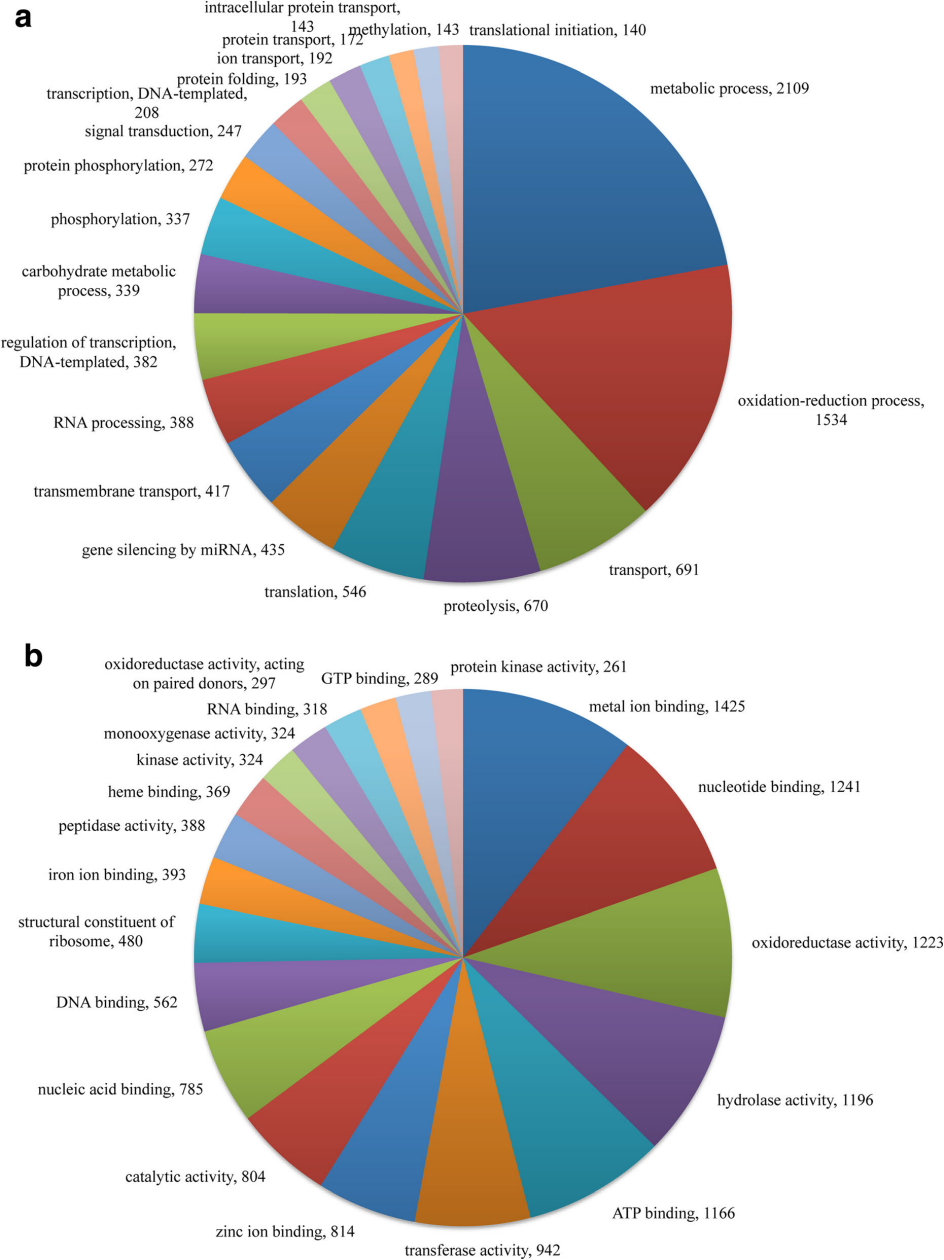
Gene Ontology (GO) assignments for FAW transcriptome, showing the 20 most-represented GO categories, presented as biological processes (**a**) and molecular functions (**b**). The number of unigenes within each GO category is shown after the comma

Annotation by Gene Ontology resulted in 1038 different processes and 9558 unigenes, and 853 different molecular functions and 13,601 unigenes (the complete databank is available upon request). Metabolic process GOs were associated with 12% of annotated contigs, followed by oxidation-reduction process, with 8% (Fig. 3A). Sequences annotated as metabolic process included mainly glutathione transferases and UDP-glucosyltransferases and UDP-glucuronosyltransferases. Oxidation-reduction process sequences included mainly cytochrome P450, and many glucose dehydrogenase (PFAM: GMC oxidoreductase) and NADH dehydrogenase sequences.

The most represented categories of GOs in molecular functions were metal-ion binding, nucleotide binding, oxidoreductase activity, and hydrolase activity (Fig. 3B). Unigenes annotated as metal-ion binding and oxidoreductase activity included several alcohol dehydrogenases, several kinds of cytochromes, including cytochrome b and cytochrome c, and many entries of cytochrome P450. Nucleotide binding included many unigenes annotated as serine/threonine protein kinase, as well as T-complex protein, ras-related protein, and multidrug resistance-associated protein, among many others. Unigenes annotated as hydrolase activity function included many peptidases (such as trypsin, esterase, serine protease, and proteasome), juvenile hormones, and serine/threonine-protein phosphatase, among others.

### Differential regulation on primary and alternative host plants

**Fig. 4:**
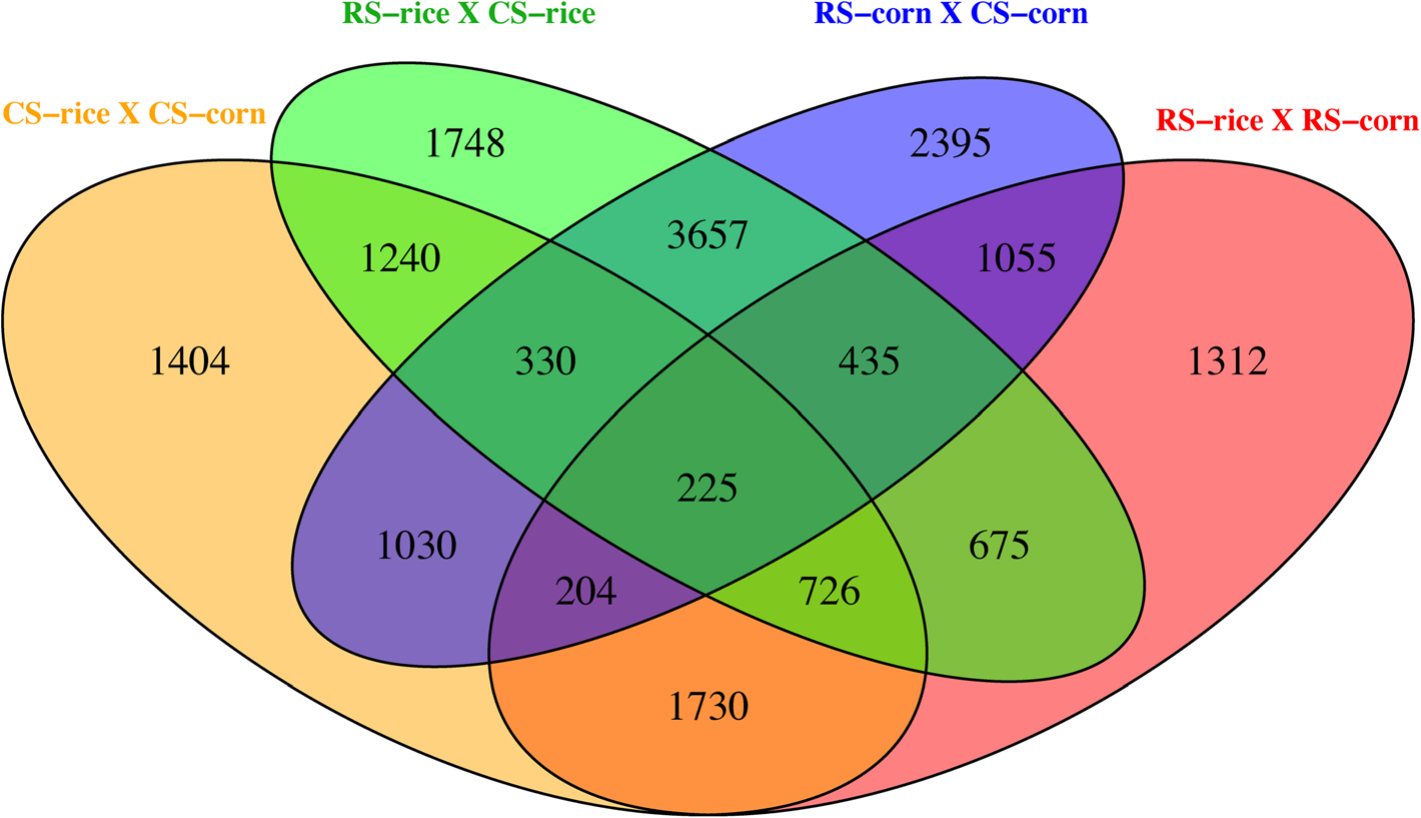
Number of at least two-fold differentially regulated genes in each comparison between pairs of feeding conditions

Comparisons of gene expression between pairs of feeding conditions revealed 225 contigs that are differentially regulated (at least two-fold) in all conditions (Fig. 4). The comparisons between the two host strains feeding on primary or alternative host plants resulted in 3657 contigs that are differentially regulated, and we considered them constitutive responses to the host plant. The main results of the two comparisons designed to investigate evolved or constitutive response of the two strains to the same host plant are described below.

**Fig. 5:**
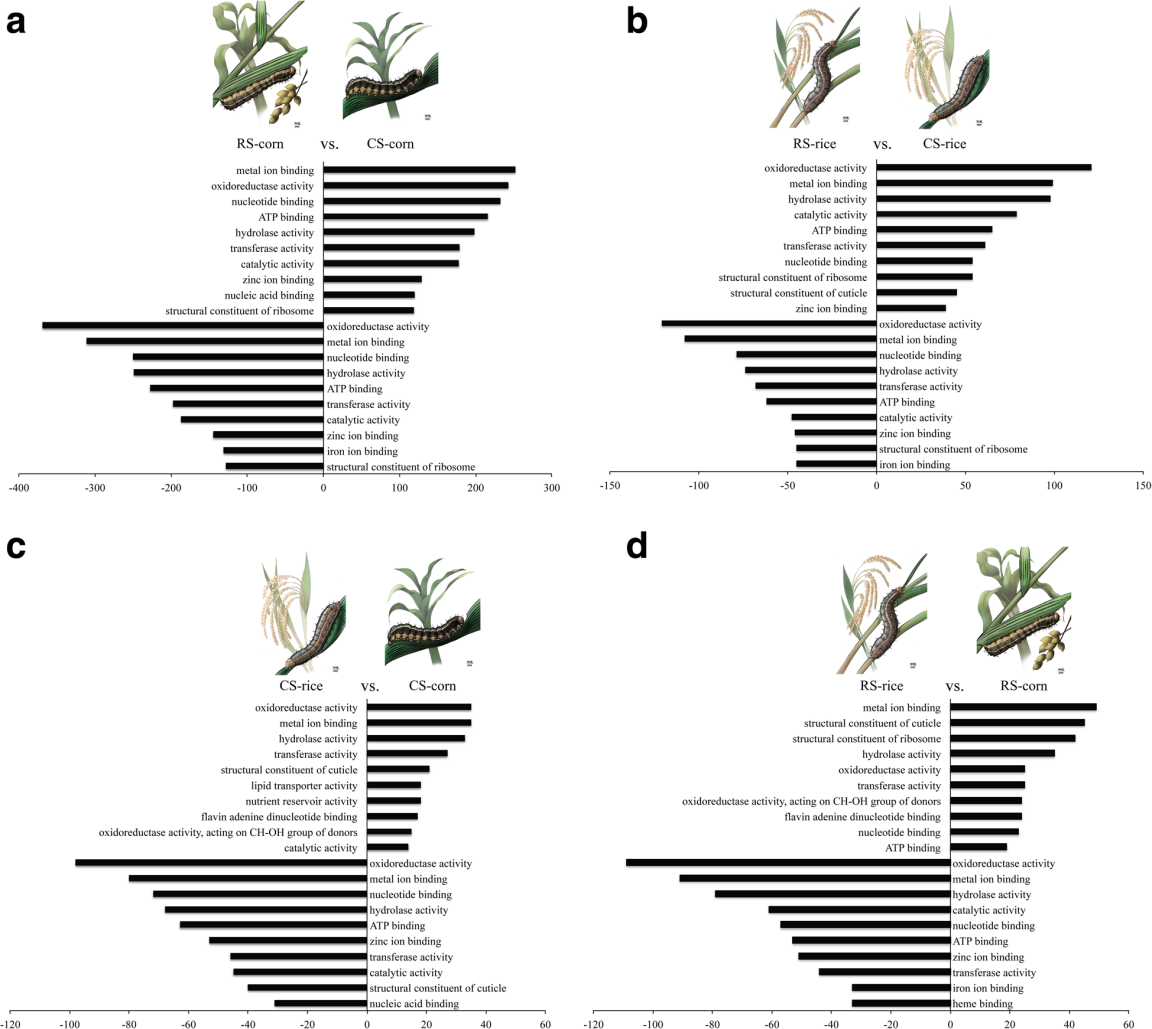
Those Gene Ontologies (GOs) are the 10 most-represented Gene Ontologies categories of up- and down-regulated contigs in each pairwise comparison presented as molecular function. **a** and **b**: constitutive response; **c** and **d**: plastic response. Up- and down-expression is a result of the comparison of the second condition in relation to the first condition. X-axis represents a different expression, and is presented on different scales for better visualization. Illustration: Dadi (www.ilustradoradadi.com)

1) **RS-corn vs. CS-corn**: A total of 2395 contigs were differentially expressed when comparing the transcriptome of the two host strains fed on corn (Fig. 4). The number of contigs differently expressed was highest in this pairwise comparison contrasted with all others (Fig. 4). Gene Ontology annotation for molecular function was assigned for 768 differentially regulated contigs; 482 contigs were up-regulated in CS and 286 were down-regulated in CS in relation to RS. The top five most differentially regulated contigs, both up- and down-regulated, include molecular functions such as metal-ion binding (GO:0046872), oxidoreductase activity (GO:0016491), nucleotide binding (GO:0000166), ATP binding (GO:0005524), and hydrolase activity (GO:0016787) (Fig. 5A). The most common unigenes annotated as metal ion-binding and oxidoreductase activity functions are cytochrome P450 (4d1-like, 4 g15, 9120, CYP4L6 and CYP6AE9), and many cytochrome b and c unigenes. Unigenes with oxidoreductase activity also included aldo-keto reductase proteins, among others. The aldo-keto reductase (AKR) superfamily comprises several enzymes that catalyze redox transformations involved in biosynthesis, intermediary metabolism, and detoxification. Substrates of the family include glucose, steroids, glycosylation end products, lipid peroxidation products, and environmental pollutants [53].

The most common unigenes with nucleotide and ATP binding functions included different subunits of the T-complex protein 1 and serine/threonine-protein kinases. Unigenes with hydrolase activity included mainly serine proteases (MEROPS: S01). Highly differentially regulated contigs also included other functions such as peptidase activity, monooxygenase activity, serine-type endopeptidase activity, and even an odorant-binding function, described as pheromone-binding protein.

2) **RS-rice vs. CS-rice**: A total of 1748 contigs were differentially regulated when both strains were fed on fresh rice leaves (Fig. 4). Six hundred fourteen (614) contigs were assigned to specific molecular functions, from which 290 were up-regulated in CS and 324 were down-regulated in CS in comparison to RS reared on rice. Again, the three most differentially regulated contigs, both up- and down-regulated, included molecular functions such as oxidoreductase activity, metal-ion binding, and hydrolase activity (Fig. 5B). The most common unigenes annotated as oxidoreductase activity and metal ion-binding functions are cytochrome P450 (e.g., 302A1, 4 g15, 9a20, CYP4L6, CYP6AE9, 6AB4, CYP332A1, CYP9A21), and many cytochrome b, acyl-CoA, aldo-keto reductase, sorbitol dehydrogenase, NADH dehydrogenase unigenes; many unigenes were annotated as zinc-finger protein under metal ion-binding function. Unigenes annotated as catalytic and ATP-binding functions were also up-regulated in RS, and include unigenes such as kynureninase, p270, serine hydroxylmethyltransferase, transketolase and C-1-tetrahydrofolate synthase (catalytic function), 26S protease regulatory, C-1-tetrahydrofolate synthase and T-complex protein 1 (ATP binding function). Down-regulated unigenes in CS (up-regulated in RS when reared on rice) include nucleotide binding and transferase activity functions; under nucleotide binding category the most common unigenes were annotated as 26S protease regulatory, ABC transporter, serine/threonine-protein kinase, T-complex protein 1, and tubulin chain. Transferase activity unigenes included mainly glutathione transferases, followed by UDP-glucosyltransferases and serine/threonine-protein kinase.

A total of 1730 contigs were differentially regulated as a response of the same strain to primary or alternative host plants, and were considered as plastic responses; 1404 contigs were differentially regulated in CS fed on corn and rice, and 1312 in RS fed on the two host plants (Fig. 4). The main results of the two comparisons designed to investigate the plastic response of each strain to the two host plants are described next:

3) **CS-rice vs. CS-corn**: A total of 381 contigs were appointed to particular molecular functions. Of these, 124 were up-regulated in CS fed on rice, and 257 were down-regulated in the same condition, in comparison to CS reared on corn. The three most differentially regulated functions, presented as up- and down-regulated, include oxidoreductase activity, metal-ion binding, and hydrolase activity (Fig. 5C). The most common unigenes annotated as oxidoreductase activity include several cytochrome P450 (e.g., 4 g15, 6AB4, 302A1, CYP6AE9, CYP4L6) and apoptosis-inducing factor. Metal ion-binding function also comprised cytochromes P450, and several zinc-finger proteins. Unigenes annotated as hydrolase activity comprised serine proteases. Up-regulated contigs also include functions such as transferase activity (mainly glutathione transferase) and structural constituent of cuticle (mainly cuticular protein RR-1 and RR-2 motifs). Down-regulated contigs also comprised nucleotide and ATP-binding functions (mostly multidrug resistance proteins).

4) **RS-rice vs. RS-corn**: A total of 420 contigs were assigned to specific molecular functions. Of these, 183 were up-regulated in RS reared on rice, and 237 were down-regulated in the same condition, in comparison to RS fed on corn. The top five most differentially up-regulated contigs include molecular functions such as metal-ion binding, structural constituent of cuticle, structural constituent of ribosome, hydrolase activity, and oxidoreductase activity (Fig. 5D). The five most differentially down-regulated contigs also include oxidoreductase activity, metal-ion binding, and hydrolase activity, and also comprised catalytic activity and nucleotide binding. The most common unigenes annotated as metal-ion binding and oxidoreductase activity include several cytochrome P450 (e.g., 9a20, 4 g15, 6AE8, CYP6AE9, CYP332A1, CYP4M5), zinc-finger proteins, sorbitol dehydrogenase, and malate dehydrogenase. Unigenes annotated as hydrolase activity comprise beta-N-acetylglucosaminidases, integument esterase, and alpha-amylases. Unigenes under the structural constituent of cuticle function include mainly cuticular proteins RR-1 and RR-2 motifs, and unigenes under the structural constituent of ribosome function comprise mainly the 60S ribosomal protein. Finally, down-regulated unigenes under catalytic-activity functions include kynurenine/alpha-aminoadipate aminotransferase and malate dehydrogenase, and those under nucleotide-binding function contain mainly a tubulin beta chain.

**Fig. 6:**
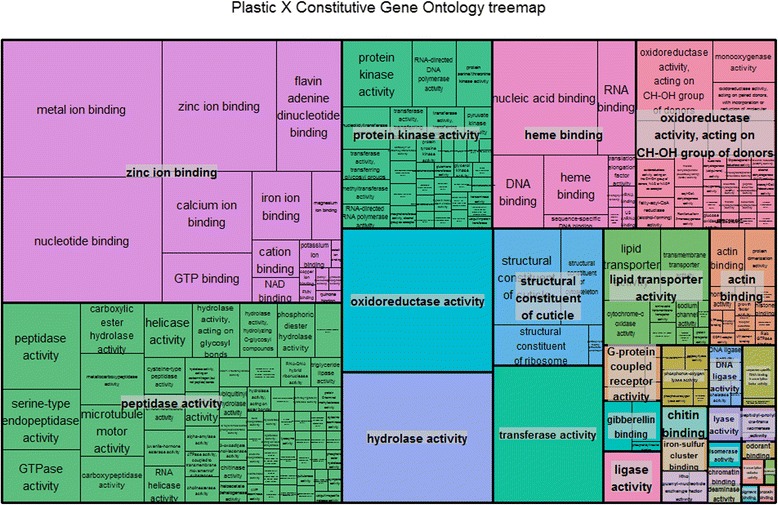
Gene Ontology (GO) annotation summarized using REVIGO of simultaneously plastic and constitutive differentially regulated genes as a response to primary and alternative host plants

Functional-ontology groups enrichment of genes that simultaneously show plastic and constitutive responses as differentially regulated contigs indicated that ca. 21% (1541 in 7219) of the plastic-response genes were also present as evolved-response contigs. These contigs include several different molecular functions (Fig. 6), and the 10 most-enriched genes in both types of comparisons are involved in oxidoreductase activity, constitution of cuticle, transport of lipids and transcription, structural integrity of a cytoskeletal structure, amino-acid attachment and synthesis of peptides, pyruvate kinase activity, and polymerase activity (Table 3). GO terms identified as significant terms have considerably large numbers of annotated genes, which gives additional confidence in the significance of our results.

**Table 3:** Ten most-enriched GO terms identified by the weight01 method implemented in topGO. Annotaded: number of genes annotated under the GO term; Significant: GOs identified as significant terms; Expected: number of interesting genes mapped to the GO term if the interesting genes were randomly distributed over all GO terms; Classic Fisher: test statistics based on gene counts [52]

GO.ID	Term	Annotated	Significant	Expected	classicFisher
GO:0016614	oxidoreductase activity, acting on CH-OH group of donors	529	31	10.32	1.6e-07
GO:0050660	flavin adenine dinucleotide binding	494	29	9.64	1.8e-07
GO:0042302	structural constituent of cuticle	263	22	5.13	1.3e-08
GO:0005319	lipid transporter activity	164	15	3.2	4.7e-07
GO:0005200	structural constituent of cytoskeleton	158	14	3.08	2.9e-06
GO:0004181	metallocarboxypeptidase activity	153	11	2.98	0.00022
GO:0016597	amino acid binding	29	4	0.57	0.00231
GO:0016743	carboxyl- or carbamoyltransferase activity	27	3	0.53	0.00112
GO:0004743	pyruvate kinase activity	14	3	0.27	0.00229
GO:0003968	RNA-directed RNA polymerase activity	12	4	0.23	6.3e-05

## Discussion

The study of the speciation process in *Spodoptera frugiperda* strains that exploit different hosts is valuable for many reasons, one of them worthy of special mention: pest-insect problems are also evolutionary problems, in the sense that it is evolution that creates genetic modification in pest populations [32]. In doing so, pest-insects can offer simpler models of study for understanding evolution and speciation in herbivorous insects.

The differences in development time of FAW host strains reared on corn and rice found here were also found in other studies, in which RS larvae required longer to pupate than CS larvae on corn [54]. Groot and collaborators [19], however, summarized several performance experiments with *S. frugiperda* reared on corn and rice, which gave variable results. In general, RS is recorded as outperforming CS on rice (under the same parameters that we measured here), while CS tended to perform better than RS when reared on corn. In our experiment, both strains showed poorer performance when fed on rice, mainly in relation to the time needed for pupation. In field conditions, taking longer to pupate can mean that the larvae are more susceptible to predation and parasitism [55], and the FAW would have an ecological advantage when feeding on corn in relation to rice. In general, performance on a new or alternative host can be lower than the performance on the native host plant [34]. Our results suggest that corn is currently the preferred host plant of *S. frugiperda*, although both rice [27] and dicots [33] have been suggested as likely ancestral host plants of the FAW.

Although we found several plastic-response genes that were also constitutive-response genes, we were not able to show that the first kind of response is overrepresented in relation to the second, as found by other studies [34]. This result could be a consequence of the high number of differentially regulated genes that we found in each rearing comparison, due to the unbiased approach that we used. Despite this, a substantial proportion of contigs are simultaneously overregulated within the same host strain feeding on different host plants and in the two host strains feeding on the same host, and we can consider that the molecular functions that they have in this insect-plant system are important as both kinds of responses. The presence of the same genes as a plastic and evolved response, as we defined here, agrees with the model proposed by West-Eberhard [12] in which the use of a new environment would arise through a plastic response, and would be eventually selected and fixed in the population by repetition of the initial stimulus and by genetic assimilation.

In summary, three molecular functions were present in all four comparisons between pairs of feeding conditions, both down- and up-regulated: oxidoreductase activity, metal-ion binding, and hydrolase activity, as also found for other insect-plant systems [30]. Oxidoreductase activity and metal-ion binding include predominantly unigenes of several kinds of cytochrome P450, involved mainly in xenobiotics metabolization [56] and zinc-finger proteins. Unigenes under hydrolase activity comprise serine proteases and trypsins, involved in protein digestion in insects, as a response to plant protease inhibitors [31, 57, 58], and sometimes used as an anti-herbivore bypass mechanism. Hydrolase activity also includes fumarylacetoacetate hydrolases, proteasome subunits, and venom serine carboxypeptidase, which has the ability to release amino acids from the C-terminus of a peptide chain, can be employed in the determination of amino-acid sequences [59], and is used as a digestive enzyme by some insects [60].

Generalist herbivores have a range of detoxifying enzymes that enable them to feed on a diversity of available host plants [31]. These enzymes include many of the unigenes described as up- and down-regulated in our comparisons, such as cytochrome P450 monooxygenases (P450s), glutathione transferases, and UDP-glycosyltransferases.

P450 enzymes were the most commonly up- and down-regulated unigenes in all of our comparisons between conditions, both as plastic and as evolved responses by the FAW strains to their primary and alternative host plants, as found in other studies [54, 61–63]. In a very comprehensive review of P450s, Feyereisen [64] stated that most transcriptomics studies have shown that one or more P450s genes were differentially regulated, but in addition to being a direct relationship, this may be a cascade effect. Cytochrome P450, or *CYP* genes, comprises one of the largest families of genes. These enzymes have a monooxygenase function, catalyzing the transfer of one atom of molecular oxygen to a substrate and reducing the other atom to water, and also show several other catalytic activities [64]. An important role of P450s in insects is related to the detoxification of xenobiotics, and P450s are involved in many events of resistance to insecticides. P450 gene expression is regulated by chemicals that allow insects to respond to new conditions directly by building a detoxification defense, and indirectly by adapting their basal metabolism, including the hormone balance, rate of development and reproduction [64]. New hosts that contain new, and possibly toxic, compounds can plastically inhibit or enhance P450 gene expression, as we found here. Among several other functions performed by P450s [65], an important function related to reproductive isolation is the role of P450 enzymes in the biosynthesis of many insect pheromones and allomones, and evidence is accumulating for pheromone catabolism by P450 enzymes [64].

Serine proteases, also highly differentiated in our comparisons, are mostly related to digestion in insects, since they produce abundant proteases for the digestion of dietary proteins [58]. Many lepidopteran larvae use serine proteases for protein digestion [31], and other studies have found that the expression of these proteases depends on the host plant [30]. These digestive enzymes allow insects to overcome plant protein-inhibitor defenses, either by overexpressing existing proteases that are not a target of the inhibitors, or by expressing new ones [58, 66]. Serine proteases, however, are associated with several non-digestive functions in insects, and often function in cascade pathways; this can occur in insect embryonic development and immune responses [67]. Serine proteases can also have a physiological function in defense against infection, when they are present in the hemolymph [67].

Plant secondary metabolites can trigger these metabolic and/or digestive responses in insects. Corn, the primary host plant tested here, contains benzoxazinoids (BXDs) or hydroxamic acids, one of the main secondary compounds in many grasses. BXDs confer resistance on herbivorous insects and pathogens because of their antifeeding, insecticidal, antimicrobial and allelopathic activities [68–70]. These compounds are present in several cereal crops such as corn, wheat, and rye, but are absent in rice, cultivated barley and oats. DIMBOA (2,4-dihydroxy-7-methoxy-1,4-benzoxazin-3-one) is the major BXD in the aerial parts of corn [71]. Although its mode of action is not fully elucidated, this hydroxamic acid inhibits digestive proteases in the midgut of larvae of the lepidopteran *Ostrinia nubilalis* (Pyralidae) [72] and *Sesamia nonagrioides* (Noctuidae) [73], suggesting that it acts as a digestive toxin [74]. When 5th instar larvae of *O. nubilalis* were fed on leaves of corn, several detoxification enzymes in the midgut, such as cytochrome b5, NADPH-cytochrome *c* reductase, NADPH oxidase and *O*-demethylase, increased activity [75]; while in in vitro tests with *S. nonagrioides*, esterases and glutathione transferases were strongly inhibited by DIMBOA [73]. Additionally, larvae of *Ostrinia furnacalis* fed on cabbage dipped in DIMBOA showed increases in the activity of cytochrome P450 monooxygenase and glutathione transferase [76]. Interestingly, although DIMBOA has a feeding-deterrent and/or toxic action for several lepidopteran pest species [68, 73], it acts as a feeding stimulant for *S. frugiperda*, and enhances FAW larval growth at low concentrations [69, 77]. All things considered, transcriptional differences in the responses of CS and RS to corn and rice leaves can be related to the presence of DIMBOA in corn, and/or to its absence in rice. Additional work with feeding of FAW with purified BXDs from corn seedlings added to an artificial diet could help to clarify this hypothesis.

## Conclusions

As expected, differentially regulated genes in all feeding comparisons involved multiple regulatory genes and processes [13]. Using a much more informative molecular approach to evaluate the unbiased transcriptome profile of *S. frugiperda*, we were able to show that phenotypic plasticity and subsequent selection in response to alternative host plants is the product of actions of many loci, with diverse molecular functions through different hierarchies, and small individual effects. Metabolism, however, is suggested as the most important function, and the variable regulation of this molecular function indicates that metabolization of foreign chemicals is among the key players in the phenotypic variation in FAW strains. From an agricultural perspective, high plasticity in detoxifying-gene families indicates the possibility of a rapid response to control substances such as insecticides.

## Abbreviations

AKR: Aldo-keto Reductase
BXDs: Benzoxazinoids
CAF: Common Assembly Format
COI: Cytochrome c Oxidase Subunit I
CS: Corn Strain
CYP: Cytochrome P450
DIMBOA: 2,4-dihydroxy-7-methoxy-1,4-benzoxazin-3-one
EST: Expressed Sequence Tag
FAW: Fall Armyworm
GO: Gene Ontology
HSP: High-scoring Segment Pair
LaCTAD: Central Laboratory of Higher Performance Technologies
NaCl: Sodium Chloride
PFAM: Protein families
RFAM: RNA families
RNA-Seq: RNA-sequencing
RPKM: Reads Per Kilobase Million
RS: Rice Strain
SQL: Structured Query Language
TAE: Tris-acetate-EDTA buffer
TMM: Trimmed Mean of M-values
